# Functional connectivity of the language area in migraine: a preliminary classification model

**DOI:** 10.1186/s12883-023-03183-w

**Published:** 2023-04-04

**Authors:** Chen Gou, Shuangfeng Yang, Qianmei Hou, Peter Rudder, Onur Tanglay, Isabella Young, Tingting Peng, Weiwei He, Liuyi Yang, Karol Osipowicz, Stephane Doyen, Negar Mansouri, Michael E. Sughrue, Xiaoming Wang

**Affiliations:** 1 Institute of Neurological Diseases, North Sichuan Medical College, Nanchong, 637000 China; 2 Department of Neurology, Affiliated Hospital of North Sichuan Medical College, Nanchong, 637000 China; 3 Omniscient Neurotechnology, Sydney, NSW 2000 Australia; 4 Shenzhen Xijia Medical Technology Company, Shenzhen, Guangdong Province 518052 China

**Keywords:** Migraine, Machine learning, Functional connectivity, Graph theory

## Abstract

**Background:**

Migraine is a complex disorder characterized by debilitating headaches. Despite its prevalence, its pathophysiology remains unknown, with subsequent gaps in diagnosis and treatment. We combined machine learning with connectivity analysis and applied a whole-brain network approach to identify potential targets for migraine diagnosis and treatment.

**Methods:**

Baseline anatomical T1 magnetic resonance imaging (MRI), resting-state functional MRI(rfMRI), and diffusion weighted scans were obtained from 31 patients with migraine, and 17 controls. A recently developed machine learning technique, Hollow Tree Super (HoTS) was used to classify subjects into diagnostic groups based on functional connectivity (FC) and derive networks and parcels contributing to the model. PageRank centrality analysis was also performed on the structural connectome to identify changes in hubness.

**Results:**

Our model attained an area under the receiver operating characteristic curve (AUC-ROC) of 0.68, which rose to 0.86 following hyperparameter tuning. FC of the language network was most predictive of the model’s classification, though patients with migraine also demonstrated differences in the accessory language, visual and medial temporal regions. Several analogous regions in the right hemisphere demonstrated changes in PageRank centrality, suggesting possible compensation.

**Conclusions:**

Although our small sample size demands caution, our preliminary findings demonstrate the utility of our method in providing a network-based perspective to diagnosis and treatment of migraine.

## Background

Migraine is a common primary headache disorder characterized by attacks of debilitating headaches [1]. Although its pathophysiology remains poorly understood, there is a suggested dysfunction of the brain in regulating pain and external stimuli, as in other chronic pain syndromes [2]. Around one-third of migraine headaches are preceded by a visual, auditory, or somatosensory aura, and a majority are associated with nausea, vomiting, and sensitivity to light. Currently, diagnostic pathological changes have not been identified, and diagnosis relies on retrospective patient reports of headache characteristics [3]. However, the heterogeneity in individual patient symptoms, along with a long list of differential diagnoses may sometimes complicate accurate diagnosis, accounting for ongoing underdiagnosis and undertreatment of this chronic condition [4, 5]. Evidently, further research is necessary to elucidate the pathophysiology of migraine and develop biomarkers for diagnosis.

The use of neuroimaging may be a potential avenue for improving diagnosis. Several studies have relied on functional magnetic resonance imaging(fMRI) to identify differences between patients with migraine and healthy controls (for reviews see: Skorobogatykh et al. [6] and Schwedt et al. [7]). Lee et al. using an independent components analysis (ICA) of migraine resting-state fMRI (rsfMRI) found an independent component that they termed the “pain matrix” (based on its labeling within Neurosynth) that included: the dorsolateral prefrontal cortex (DLPFC), anterior insula, anterior cingulate cortex (ACC), thalamus, precuneus, supramarginal gyrus, planum temporale, premotor cortex, and cerebellum [8]. While they do not label the network affiliations of these regions of the pain matrix, their findings are comparable to Coppola et al., who used a similar analytic approach and found that portions of the default mode network (DMN) and central executive network (CEN) (which overlap with Lee’s Pain Matrix) express differential connectivity to healthy controls (HCs) [9]. Research also suggests that functional connectivity (FC) among regions along the nociceptive and somatosensory pathways is disrupted during the different phases of migraine headache [6, 7, 10–12]. In a series of recent papers, Meylakh and colleagues have been utilizing rfMRI to elucidate the underlying mechanisms of brain dysfunction in the context of migraine; revealing that brainstem pain areas are anomalously regulated (via the descending pain modulatory pathway) by higher cortical regions [13–16]. While the mechanisms and specifics are still unknown, the literature is converging on a consensus regarding the network associations with migraine, including the DMN, CEN, sensorimotor network (SMN), and limbic system [17–20].

Despite these findings, definitive fMRI biomarkers have not yet been identified, particularly due to the heterogeneity in study methods. There has also been a scarcity of studies which integrate fMRI findings into machine learning models to develop clinical tools for diagnosis. Zhang et al. combined functional and structural MRI to distinguish patients with migraine from controls with 83.67% accuracy [21]. Chong et al. utilized the FC of predefined pain-related regions to discriminate migraine sufferers from controls [22]. Their model achieved an accuracy of 96.7% in patients with migraine with a longer disease duration (> 14 years), and 82.1% in those with a shorter disease duration (< 14 years). These findings are exciting, however, the existing machine learning attempts rely on unspecific anatomical templates, manifesting in the use of terms such as DLPFC or ACC. Given each of these regions are now known to comprise of several smaller regions [23], it is currently difficult to adopt findings as objective biomarkers for intervention. Therefore, more rigorous, and sophisticated methods are required to examine changes in FC and utilize these in diagnosis and treatment.

In the present study, we used FC and graph theory analysis with a sophisticated machine learning technique on a cohort of patients with migraine and healthy controls. The machine learning classification was coupled with a recently described feature importance derivation method to identify disturbances in specific brain regions and large-scale networks underlying migraine. We hope our methodology can provide further insight into the pathophysiology of migraine and offer targets to improve patient outcomes.

## Methods

### Patient cohort

Altogether 35 patients with migraine and 20 healthy controls (HCs) from the Department of Neurology of the Affiliated Hospital of North Sichuan Medical College were recruited from January 2021 to January 2022 for this study. Two patients with migraine and three HCs had to be excluded due to head movements during fMRI acquisition, and two patients with migraine were excluded due to previous imaging data suggesting cerebrovascular malformation. 31 patients with migraine and 17 HCs were finally included in the study. The subjects met the criteria for episodic with or without aura migraine as classified as per the International Classification of Headache Disorders, 3rd edition [24]. HCs had no history of migraine or other headaches, no family history of migraine. All participants were Chinese, right-handed, and between 18 and 50 years old. Patients had no migraine 72 h prior to the scan and no symptoms of developing one during or 24 h after the scan. Neither migraineurs nor HCs were taking medication, including preventative medication for migraine. Female subjects were not in the menstrual phase of their cycle (days 1–7). All subjects underwent a general physical and neurological examination. Participants completed the Hamilton Depression Rating Scale (HAMD), Hamilton Anxiety Rating Scale (HAMA), the Migraine Specific Quality of Life survey (MSQ), and the Visual Analog Scale (VAS),and subsequently underwent Diffusion Weighted Imaging (DWI) and rsfMRI.

Exclusion criteria for all subjects included: (i) other primary headaches or secondary headache; (ii) definite history of neuropsychiatric diseases or internal disease; (iii) drug, alcohol and tobacco addiction; (iv) pregnancy or lactation; (v) steroid, psychiatric, and immunosuppressive drug users; (vi) MRI contraindication.

Patients with migraine completed the HAMD, HAMA, the MSQ, and the VAS. All participants subsequently underwent DWI, and rfMRI. The study received ethics committee approval from the Affiliated Hospital of North Sichuan Medical College. All participants signed written informed consent. The study has been performed in accordance with the Declaration of Helsinki.

### Image acquisition

All examinations were performed on Signa Excite HD 3.0 T superconducting MR system (GE, Milwaukee, WI, USA) with a 32-channel phased array head coil. During MRI scanning, participants were instructed to rest quietly, stay awake and breathe smoothly until the end of the examination. The gap between the subject’s head and the coil was filled with sponge to minimize head movement.

T1-weighted image scan parameters included: echo time (TE) = 3.2 ms, repetition time (TR) = 8.2 ms, flip angle = 12°, matrix = 256x256, 1 mm slice thickness. Functional MRI scan parameters: echo-planar imaging (EPI) sequence, TR = 2,000 ms, TE = 30 ms, flip angle = 90°, field of view is 24 cm×24 cm, matrix = 64 × 64, slice thickness = 4 mm, slices = 33.

Diffusion weighted imaging parameters included: 20 contiguous slices, slice thickness = 5 mm, FOV = 220 × 220 mm, matrix = 128 × 128 mm, TR = 6000 ms, TE = 114 ms, acquisition NEX = 2 partial Fourier, 64 diffusion directions with b-value = 1000 s/mm2, and one image with no diffusion weighting (b = 0 s/mm2), bandwidth = 250 Hz/pixel. Acquisition time was 19.16 min per DWI scan.

### Diffusion weighted imaging (DWI) preprocessing

The DWI images were processed using the Infinitome software [25], which employs standard processing steps in the Python language. Initially, the diffusion image was resliced to ensure isotropic voxels and motion correction was performed using a rigid body registration algorithm to a baseline scan. Slices with excess movement, defined as DVARS > 2 sigma from the mean slice were eliminated [26]. The T1 image was skull stripped using the HD-BET software [27], which was inverted and aligned to the DWI image using a rigid alignment, and used as a mask to skull strip the DWI image. Next, gradient distortion correction was performed by applying a diffeomorphic warping registration method between the DWI and T1 images. The fiber response function was then estimated using constrained spherical deconvolution and deterministic tractography was performed with uniform seeding, with four seeds per voxel. This manifests in about 300,000 streamlines per brain.

### Structural connectivity based parcellation

Identifying connectivity changes at an anatomical level requires an atlasing scheme to parcellate the brain. While several schemes are available, many rely on healthy cortices for parcellation. Furthermore, most available atlases parcellate a given scan based on the group average of these healthy cohorts, neglecting the possible impacts of gyral variation or morphological differences brought on by pathology. In order to mitigate this, we adopted a machine-learning based method to create subject specific versions of the Human Connectome Project Multimodal Parcellation (HCP-MMP1) atlas [23], which has been described elsewhere [28]. This method relies on a machine learning model which is trained using DWI data from 178 healthy controls obtained from the SchizConnect database, processed as above, to learn the structural connectivity pattern between voxels included within the 379 parcels of the HCP-MMP1 atlas. In order to parcellate the DWI scans in the current study, the unaltered HCP-MMP1 atlas was initially warped onto each brain and the trained machine learning model was applied to each subject to re-appoint voxels located at the endpoint of tractography streamlines to their most likely parcellation based on the structural connectivity vectors. This reparcellates the voxels, creating a subject specific version of the HCP-MMP1 atlas with 180 cortical parcels and 9 subcortical structures per hemisphere, along with the brainstem as one parcel.

The network affiliation of each HCP parcels was based on the automatic mapping provided by the Infinitome software, which itself is based on previous meta-analyses exploring each large-scale network. The networks included in this template were the core networks described by Yeo et al. [29], the CEN, DMN, Dorsal Attention Network (DAN), Limbic Network (LN), Salience Network (SN), Sensorimotor Network (SMN), and the Visual Network (VN), along with several networks which are either part of the extended versions of the core networks, or additional networks described in the literature, including the Accessory Language and Language Networks (part of the extended DMN), Auditory System (part of the SMN), Multiple demand network, and the Ventral Attention Network (VAN).

### Resting-state fMRI pre-processing steps

The rfMRI images were processed using standard processing steps including: (1) motion correction on the T1 and BOLD images using a rigid body alignment, (2) elimination of slices with excess movement (defined as DVARS > 2 sigma from the mean slice), (3) skull stripping of the T1 image using a convolutional neural net (CNN), which is inverted and aligned to the resting state bold image using a rigid alignment, and used as a mask to skull strip the rfMRI image, (4) slice timing correction, (5) Global intensity normalization, (6) gradient distortion correction using a diffeomorphic warping method to register the rfMRI and T1 images, (7) High variance confounds are calculated using the CompCor method [30]; these confounds as well as motion confounds are regressed out of the rfMRI image, and the linear and quadratic signals are detrended. Note this method does not perform global signal regression, (8) spatial smoothing is performed using a 4 mm full width half maximum (FWHM)Gaussian kernel. Functional connectivity analysis was performed in native space. The personalized atlas created in previous steps is registered to the T1 image, and grey matter atlas regions are aligned with the grey matter regions in each subject’s scans. Thus, it is ideally positioned for extracting a BOLD time series, averaged over all voxels within a region, from all 379 regions. The Pearson correlation coefficient is calculated between the BOLD signals of each unique area pair (self to self-inclusive), which yields 143,641 correlations.

### Machine learning classification and feature extraction using the hollow-tree Super (HoTS) method

Machine learning was used to model the diagnostic group of each participant based on the pairwise functional correlation between the 379 regions of each individual’s brain atlas. A boosted trees approach, the Extreme Gradient Boosting (XGboost) ensemble in Python [31], was used to fit the model. This approach provides a superior prediction ability than single trees.

In order to identify the features the model relied on the most to make its predictions, we utilized a recently developed feature extraction technique, Hollow Tree Super (HoTS) described elsewhere [32]. Briefly, HoTS linearizes decision trees in order to provide directional feature importance coefficients. This technique enables the extraction of feature importance in cases with a large number of features, as in the parcellated human brain, and provides the ability to derive a scale to the contribution of each feature. Consequently, for each model we attained a list of FC features, corresponding to pairwise HCP parcels, along with an indication of their impact on the overall model. The XGboost models were tuned by using a system developed by Omniscient Neurotechnology. The learning rate, the random seed initialisation value, the minimum tree depth for prediction were tested. The XGboost models were also tuned using a distribution of models with different performance metrics obtained from this process in a relatively short amount of time due to parallelisation. Furthermore, the XGboost models were also tuned using cross-validation methods to ensure that the hyperparameters were not being optimised just for the given training and test data splits. This is a form of overfitting, which was neutralised with 5-fold cross-validation. Each model was evaluated with the mean area under the receiver operating characteristic curve (AUC-ROC). For each model, we produced a network-based plot of the contribution of each large-scale brain network to the model, along with a SHAP plot of the top 20 features contributing to the model. The network-based plots were produced by aggregating the SHAP contribution values of each brain region to the model by their network affiliation [33]. Note that these plots therefore do not necessarily reflect intra-network connectivity. Each SHAP plot provides a list of features in descending order of importance, along with their impact on the model along the x-axis. The colour of each point indicates whether a high (red), or low (blue) value of that feature is associated with the model.

### Median absolute deviation outlier detection

In order to find outliers in FC in the migraine group, we calculated the median and median absolute deviation (MAD) within the FC of healthy controls. We then determined for each migraine patient whether FC was an outlier, based on a threshold MAD of 3 or more. The number of anomalies within each network were then counted and the mean calculated.

### PageRank centrality analysis

In order to investigate changes in centrality and identify hubopathy within our sample, we calculated weighted PageRank centrality from the tractography adjacency matrix of the number of streamlines connecting the 379 regions. PageRank centrality is a measure of the importance of a given brain region within a network, based on its influence on the topography of information flow in the brain. PageRank considers a region with a high number of influential connections important, however biases against connections which are endpoints [34]. Weighted PageRank centrality was calculated using the NetworkX module in Python [35]. The raw centralities were converted into a rank from 1, signifying the highest centrality, to 379, the lowest centrality, for each subject. The median rank for each region was calculated in the healthy cohort and set as the default listing order for parcellations, and this order was compared to the order in migraineurs.

### Statistical analysis

Nominal demographic data were compared between patients with migraine and controls using a Chi-squared test, while continuous variables were compared using a Mann-Whitney U test. All analyses were performed on R version 4.1.0.

## Results

### Subject demographics

Table 1 highlights the demographic characteristics for the entire cohort. The median age was 25 in patients with migraine and controls. There was a significant association between sex and diagnostic group (*Χ*^2^(1) = 3.9, *p* = 0.049). 84% of the migraine cohort was female, while the control group had an even distribution of males and females (due to the convenience sampling method for healthy controls; and clinical cohort enrollment for migraineurs) we could not sex match the groups, however, sex was included as a covariate in all models). Only 9 patients with migraine (29.0%) had migraines with aura. Median disease duration for the patients with migraine was 6 years (IQR = 4). All subjects were without depression and anxiety. The median MSQoL score of migraine patients was 62 (15.5), lower than HCs. The patients had a moderate pain intensity,

**Table 1:** Demographic and clinical characteristics for the entire cohort

Demographic	Migraineurs (*n* = 31)	Healthy Controls (*n* = 17)	*p* value
**Sex F/M (%)**	26/5 (83.9/16.1)	8/9 (47.1/52.9)	0.049
**Median age (IQR)** ***years***	25 (8)	25 (3)	0.429
**Migraine with aura (Yes/No) (%)**	9/22 (29.0/71.0)		
**Median Disease Duration (IQR)** ***years***	6 (4)		
**Attack per month**	2(1–5)		
**Median HDRS17 score (IQR)**	6 (3.5)		
**Median HAM-A score (IQR)**	6 (6)		
**Median MSQoL score (IQR)**	62 (15.5)		
**Median VAS score (IQR)**	5 (2.5)		

*HDRS17*, Hamilton Depression Rating Scale (0–52)

*HAM-A*, Hamilton Anxiety Rating Scale (0–30)

*MSQoL*, Migraine Specific Quality of Life (25–100)

*VAS*, Visual Analog Scale (0–10)

### FC differences in the language network may differentiate patients with migraine from controls

**Fig. 1:**
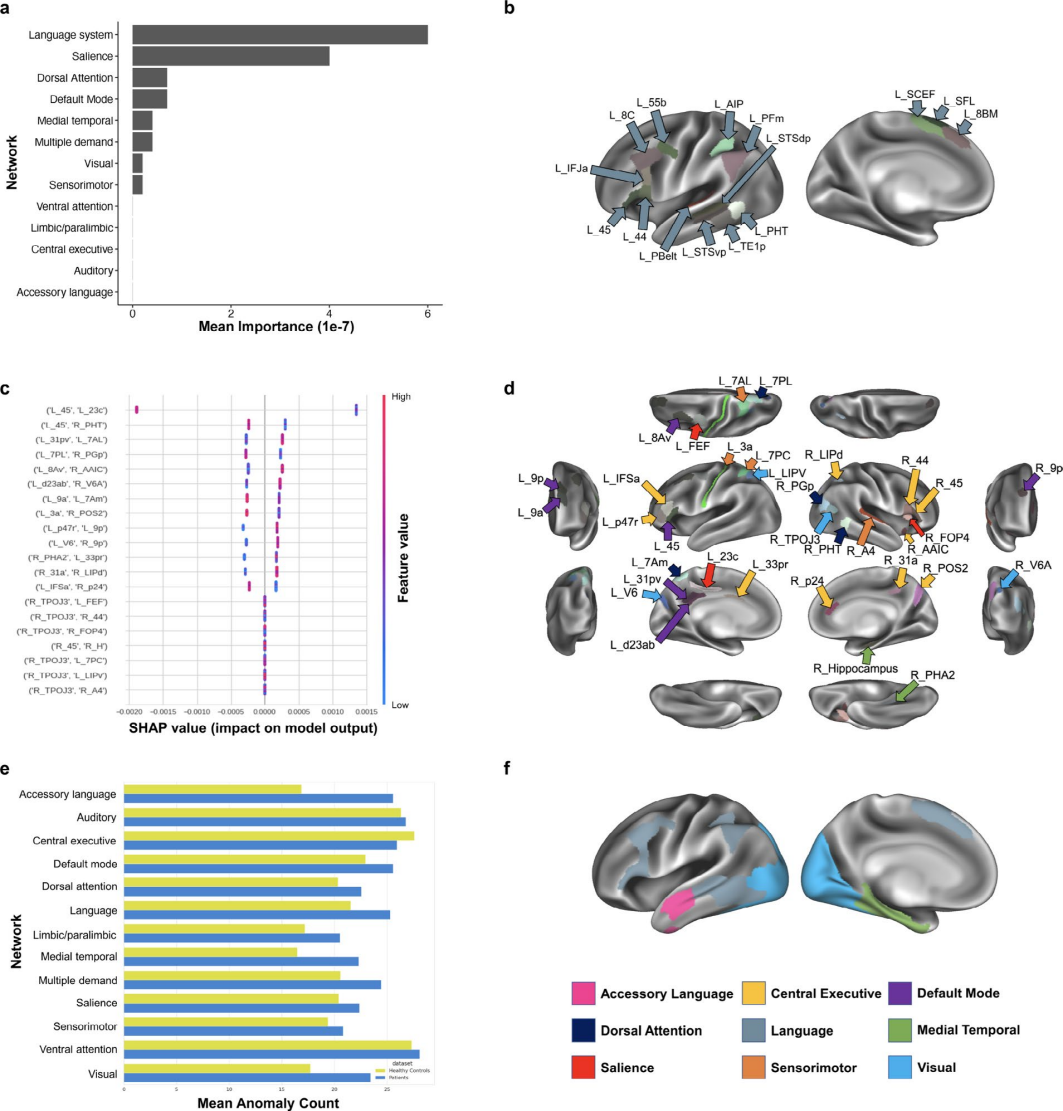
**(a)** Mean importance of each network’s contribution to the model classifying patients with migrainefrom controls based on functional connectivity. **(b)** A graphical representation of the Language network. **(c)** A SHAP plot demonstrating parcel-based feature importance in the model classifying patients with migrainefrom controls. Each feature represents pairwise functional connectivity between two regions. The features are listed in descending order of importance. The horizontal axis provides an indication of each feature’s impact on the model, and the color of each point represents whether a high or low value of the given feature is associated with its impact. **(d)** A graphical representation of the top 20 features whose functional connectivity contributed to the machine learning model’s classification. The colors of each arrow signify the network to which each parcellation belongs, with a key provided at the bottom right corner of the figure. In this representation, the language network has been classed within the Default Mode Network (purple), its primary network. **(e)** The graph represents the mean number of anomalies detected through median absolute deviation within each network, in patients with migraineand controls. **(f)** A graphical representation of the Accessory Language, Language, Medial Temporal, and Visual networks on a left sided brain. The network corresponding to each color has been provided in the key in the bottom right corner of the Fig. 31a, area 31 anterior; 31pv, area 31 posterior ventral; 33pr, area 33 prime; 7AL, lateral area 7 A; 7Am, medial area 7 A; 8Av, area 8 A ventral; 9a, area 9 anterior; 9p, area 9 posterior; A4, auditory 4 complex; AAIC, anterior agranular insular complex; AIP, anterior intraparietal area; d23ab, area dorsal 23 a + b; FEF, frontal eye fields; FOP4, frontal opercular area 4; LIPd, area lateral intraparietal dorsal; LIPv, area lateral intraparietal ventral; p24, area posterior 24; p47r, area posterior 47r; PBelt, parabelt complex; PFm, area PFm complex; PHA2, parahippocampal area 2; POS2, parieto-occipital sulcus area 2; SCEF, supplementary and cingulate eye field 2; SFL, superior frontal language area; STSdp, superior temporal sulcus dorsal posterior; STSvp, superior temporal sulcus ventral posterior; TE1p, area TE1 posteiror; TPOJ3, area temporoparietooccipital junction 3; V6, sixth visual area; V6A, sixth visual area A

Our model differentiating migraine from healthy controls initially achieved a mean AUC of 0.68 ± 0.13, which increased to 0.86 following hyperparameter tuning. At a network level, parcels belonging to the language network had the greatest contribution to the model’s classification (Fig. 1a,b). At a region level, a low FC between left area 45 (of the language network) and left area 23c (of the salience network) had the greatest impact on the model output (Fig. 1c,d). The rest of the features in the top 20 had a significantly smaller impact on model output, however, the majority of them were DMN regions, including the left area 31p ventral (31pv), left area 8Av, left area dorsal d23 a + b (d23ab), left area 9 anterior (9a), left area 9 posterior (9p), right area 9p, and right parahippocampal area 2 (PHA2); or CEN regions, including the right anterior agranular insula complex (AAIC), right parieto-occipital sulcus area 2 (POS2), left area posterior 47r (p47r), left area 33 prime (33pr), right 31 anterior (31a), right area lateral intraparietal dorsal (LIPd), left area inferior frontal sulcus anterior (IFSa), and right area posterior 24 (p24), though these were ranked lower.

Next, the MAD analysis showed the greatest number of deviations in the migraine group in the accessory language network, the language network, the medial temporal system, and visual system (Fig. 1e,f).

### PageRank centrality revealed contralateral changes in migraine

**Fig. 2:**
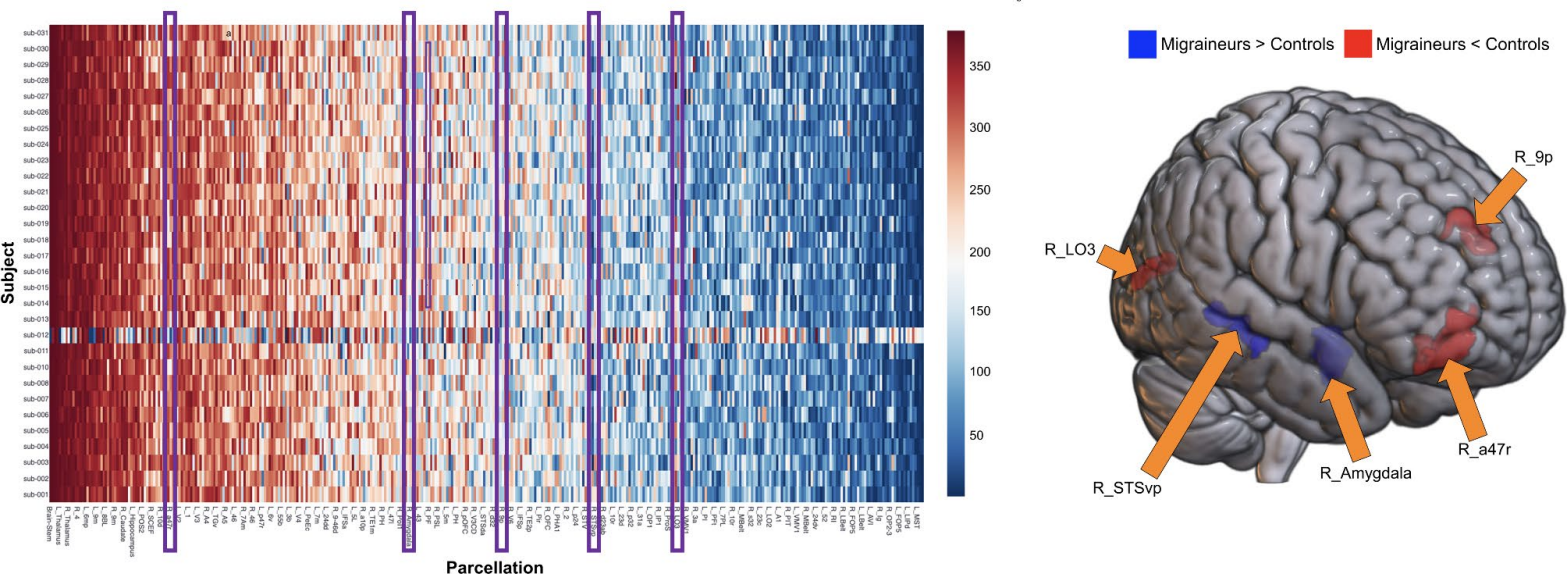
**(a)** A heatmap of PageRank centrality of all 379 brain regions in migraine patients, ordered by the median PageRank centrality of healthy controls to identify deviations. deviations in patients with migraine from the median PageRank centrality of controls for all 379 brain regions. Each row represents a migraine subject, while each column is a brain region. Blue represents a lower PageRank centrality, while red represents a higher PageRank centrality. The purple boxes represent five brain regions which showed deviations in migraine subjects compared to controls: right LO3, right STSvp, right 9p, right Amygdala, and right a47r. **(b)** The five brain regions showing deviations in PageRank centrality are shown on a brain for anatomical reference. The red regions represent areas where migraine sufferers had a lower PageRank centrality than controls, while blue represents regions where migraine sufferers had a higher PageRank centrality than controls

PageRank centrality in migraine patients showed shifts from the median PageRank in controls in the right area lateral occipital 3 (LO3), right superior temporal sulcus ventral posterior (STSvp), right 9p, right Amygdala, and right area anterior 47 rostral (a47r) (Fig. 2a,b). The right STSvp and right amygdala demonstrated higher PageRank centrality in most patients with migraine than controls, whereas the right LO3, right 9p, and right a47r demonstrated lower centrality. All the regions demonstrating a shift in PageRank centrality were in the right hemisphere. The PageRank centrality ranks of one subject, subject 12, differed significantly from both the patient and control ranks.

## Discussion

The present study investigated the ability of a gradient boosted trees approach in differentiating patients with migraine from controls, and derived brain regions and networks which accounted for the classification differences in each case. Our findings indicate that FC differences in the language network were most predictive of diagnostic grouping, though the accessory language areas, the limbic system and sensory systems also demonstrated deviations in FC compared to controls. Furthermore, our PageRank analysis indicated that there may be analogous contralateral changes in migraine patients, which could potentially be harnessed as a treatment target. Collectively, these data highlight the utility of our methodology to explore the connectomic disturbances underlying migraine. Through validation, our findings may be used to further elucidate migraine’s pathophysiology and develop diagnostic and therapeutic modalities to improve patient outcomes.

### Interactions of the default mode and salience networks in migraine

The top feature contributing to our model’s classification was a low correlation between the left area 45, part of the default mode network [36], and left area 23c, part of the salience network [36]. Area 23 has previously been shown to be activated in response to acute pain [37], and our results may therefore be pointing to altered pain perception in patients with migraine. A reduction in the thickness of the posterior cingulate cortex, a region known to be involved in nociception and chronic pain, has been associated with migraine improvement [38]. Furthermore, temporal fluctuations in the connectivity of the salience network has previously been associated with migraine [39], and the insula, a core part of the salience network exhibits altered connectivity in migraine patients [40–42]. The salience network has previously been proposed to be responsible for switching between the introspective default mode network, and the central executive network [43]. Our findings may be pointing to alterations in this mechanism, which may be responsible for heightened awareness of pain in migraine. Promoting connectivity between these two networks through non-invasive stimulation may, therefore, provide a therapeutic option, though further studies must examine whether this association exists in larger cohorts.

### Migraine and language network changes

Interestingly, the language network was most associated with our model’s classification of patients with migraine from controls. This can be attributed to the contribution of left area 45, given it was part of the top two features contributing to the model at the individual region level. To our knowledge, no previous studies have described the language network in migraine, though this relationship may be explained by the function of this network. It should also be noted that dysphasia is not uncommon during migraine. The language network, and the accessory language network, which also demonstrated distinct connectivity in migraine, are not part of the core networks described in other studies, and are often part of extended network schemes, which are subdivisions of other networks. Some authors in fact consider these networks part of the extended default mode network [36], though the individual network affiliations of each region differs. The specific regions of the HCP atlas comprising the language network in the Infinitome scheme include left area 55b, left area 8 C, left area 44, left area 45, left area 8BM, left area IFJa, the left anterior intraparietal area (AIP), left area PFm complex (PFm), left superior frontal language area (SFL), left supplementary and cingulate eye field (SCEF), the left parabelt complex (PBelt), left STSdp, left STSvp, left area TE1 posterior (TE1p), and left area PHT, while the accessory language area includes left STSda, left TE1a, left TGv, and left STSva. These networks comprise regions such as the superior temporal area and the temporal pole, which are sites of multisensory integration. The superior temporal sulcus integrates visual, somatosensory, and auditory information [44], whereas the temporal pole is a region for integration of auditory, olfactory and somatosensory stimuli [45]. The temporal pole has been implicated in migraine, with several studies revealing cortical thinning, atypical resting-state FC, and hyperexcitability of the temporal pole in patients with migraine compared to healthy controls [46–49]. Furthermore, left area 45, the pars triangularis, was also highlighted in our study as one of the main features contributing to the model’s classification. Once again, a crucial part of the language network as one of the constituents of Broca’s area, the pars triangularis has been shown to play a role in pain empathy, and patients with migraine have shown altered FC in the region of the inferior frontal gyrus [50–54]. Together, these findings suggest that the language network may be an important locus in migraine, and may be mediated by deficits in multisensory integration resulting in altered pain perception. It is also worth noting that a previous population study has demonstrated that patients with migraine had impaired verbal ability and language reception at ages three to 13 prior to the development of headache [55]; whereas another study showed patients with migraine performed worse in several domains of the MoCA, including language compared to healthy controls [56]. It is however important to again note that all of these brain regions which are involved in language are not solely language areas. For example, area 45 is involved in sensorimotor learning and integration [57], while the left inferior frontal gyrus in general has shown involvement in response inhibition [58, 59], a deficit known to persist even outside of attacks in migraine [60], and one which parallels our hypotheses regarding the interaction between the DMN and SN in the previous section. The networks highlighted in our model also depend on the networks ascribed to each region. This was performed manually and based on the literature, as we did not perform task-based fMRI. Different studies may ascribe regions to different networks, though the affiliations provided by the Infinitome software rely on several meta-analyses and along with a recent comprehensive analysis of network affiliation specifically based on the Glasser atlas [36]. Therefore, while further studies are necessary to elucidate the nature of the relationship between language and migraine, it is nonetheless interesting that several regions known to have a role in language were highlighted in our study.

### Alterations in multiple networks underlie migraine

The visual network and the medial temporal region also demonstrated deviations in FC in migraine. The visual network has been strongly linked to migraine. Recently, Huang and Wilkins demonstrated that the distribution of the visual network was altered in migraine patients and demonstrated lateralization [61]. In another study, there was enhanced FC between the thalamus and several parts of the visual cortex, suggesting aberrant projections from the thalamus to the visual network [62]. These changes may underlie the visual symptoms associated with migraine, both with and without aura. Furthermore, the medial temporal lobe has been associated with nociception in chronic pain, with a meta-analysis demonstrating reduced activation in the right anterior hippocampus in patients with migraine [63]. A recent study has also demonstrated that mesial temporal sclerosis on MRI is a common finding in patients with migraine without a history of epilepsy, suggesting damage as a result of migraine [64]. These changes demonstrate that migraine is a complex multi-network condition which may require a whole-brain approach for targeted therapies. Methods such as ours may be promising in identifying potential targets.

### Harnessing compensatory changes in migraine

While the left sided language networks accounted most for the FC -based classification, right sided regions demonstrated the greatest amount of deviation in PageRank centrality, a measure of hubness based on structural connectivity. Regions which demonstrated higher PageRank in migraineurs, including the right STSvp, and right amygdala, may be demonstrating compensatory changes. The potential changes in the left superior temporal region in the context of the language network has been previously discussed, however, the amygdala is also a key region of nociceptive and emotional processing, and has demonstrated FC changes in migraine [65]. One study in fact demonstrated that these changes were lateralized to the left, which may explain the increased hubness of the right amygdala in our dataset [20]. This may however also be due to possibly different functions of the amygdala bilaterally, and further studies are necessary to explain these findings. Nonetheless, these regions may potentially provide targets for diagnosis and interventions such as transcranial magnetic stimulation (TMS) to treat migraine.

### Limitations

Our study has several key limitations. Our small sample size limits the generalizability of our findings, and larger prospective studies and replication in independent cohorts is necessary to substantiate our results. In addition, given our small sample size, we were unable to conduct sub-analyses to compare migraine with and without aura, which should be a goal in future studies. We were also unable to combine structural and FC data as our sample was insufficient. Multimodal data may however improve the accuracy of models and provide further directions to improve diagnosis and treatment options in migraine. Finally, the model presented in the study has not yet been applied to other types of headache or pain patients.In future, we will conduct comparative studies on different types of headache, which may have guiding significance for the differential diagnosis of migraine.

## Conclusions

Here, we utilize an explainable machine learning approach to analyze resting state FC data in a sample of patients with migraine, and a healthy control comparison group. We find that FC of the language network is most indicative of migraine status, however, other networks, including accessory language, demonstrate distinguishable characteristics. Additionally, we demonstrate a shift in hubness across the brain, with right hemisphere regions showing increased hubness in migraineurs; a potential marker and target for reorganization. While this study is too limited to define reliable biomarkers of migraine, it does suggest the possibility of aberrant brain organization across higher order cognitive networks, and extends the focus beyond the abnormal processing of pain -- to abnormal brain organization.

## Data Availability

The datasets generated during the current study are not publicly available due to privacy or ethical restrictions but are available from the corresponding author on reasonable request.

## Abbreviations

MRI: Magnetic Resonance Imaging
HoTS: Hollow Tree Super
fMRI: Functional Magnetic Resonance Imaging
rfMRI: Resting-state fMRI
DLPFC: Dorsolateral Prefrontal Cortex
ACC: Anterior Cingulate Cortex
DMN: Default Mode Network
DMN: Central Executive Network
FC: functional Connectivity
SMN: Sensorimotor Network
HAMD: Hamilton Depression Rating Scale
HAMA: Hamilton Anxiety Rating Scale
MSQ: Migraine Specific Quality of Life survey
VAS: Visual Analog Scale
DWI: Diffusion Weighted Imaging
HCP-MMP1: Human Connectome Project Multimodal Parcellation
DAN: Dorsal Attention Network
